# Recombinant Live Attenuated Influenza Vaccine Viruses Carrying Conserved T Cell Epitopes of Human Adenoviruses Induce Functional Cytotoxic T Cell Responses and Protect Mice against both Infections

**DOI:** 10.3390/vaccines8020196

**Published:** 2020-04-24

**Authors:** Irina Isakova-Sivak, Victoria Matyushenko, Ekaterina Stepanova, Anastasia Matushkina, Tatiana Kotomina, Daria Mezhenskaya, Polina Prokopenko, Igor Kudryavtsev, Pavel Kopeykin, Konstantin Sivak, Larisa Rudenko

**Affiliations:** 1 Institute of Experimental Medicine, 197376 Saint Petersburg, Russia; matyshenko@iemspb.ru (V.M.); fedorova.iem@gmail.com (E.S.); anastasiia.evsina@gmail.com (A.M.); kotomina@iemspb.ru (T.K.); dasmez@iemspb.ru (D.M.); prokkopenko@mail.ru (P.P.); igorek1981@yandex.ru (I.K.); pmkopeikin@mail.ru (P.K.); rudenko.lg@iemspb.ru (L.R.); 2 Smorodintsev Research Institute of Influenza, 197376 Saint Petersburg, Russia; konstantin.sivak@influenza.spb.ru

**Keywords:** human adenovirus, live attenuated influenza vaccine, viral vector, conserved epitopes, T cell-based vaccine, bivalent vaccine

## Abstract

Human adenoviruses (AdVs) are one of the most common causes of acute respiratory viral infections worldwide. Multiple AdV serotypes with low cross-reactivity circulate in the human population, making the development of an effective vaccine very challenging. In the current study, we designed a cross-reactive AdV vaccine based on the T-cell epitopes conserved among various AdV serotypes, which were inserted into the genome of a licensed cold-adapted live attenuated influenza vaccine (LAIV) backbone. We rescued two recombinant LAIV-AdV vaccines by inserting the selected AdV T-cell epitopes into the open reading frame of full-length NA and truncated the NS1 proteins of the H7N9 LAIV virus. We then tested the bivalent vaccines for their efficacy against influenza and human AdV5 in a mouse model. The vaccine viruses were attenuated in C57BL/6J mice and induced a strong influenza-specific antibody and cell-mediated immunity, fully protecting the mice against virulent influenza virus infection. The CD8 T-cell responses induced by both LAIV-AdV candidates were functional and efficiently killed the target cells loaded either with influenza NP_366_ or AdV DBP_418_ peptides. In addition, high levels of recall memory T cells targeted to an immunodominant H2^b^-restricted CD8 T-cell epitope were detected in the immunized mice after the AdV5 challenge, and the magnitude of these responses correlated with the level of protection against pulmonary pathology caused by the AdV5 infection. Our findings suggest that the developed recombinant vaccines can be used for combined protection against influenza and human adenoviruses and warrant further evaluation on humanized animal models and subsequent human trials.

## 1. Introduction

Acute respiratory viral infections (ARVIs) are one of the leading causes of mortality and morbidity worldwide [1], and children often experience several episodes a year. In children, clinical presentation can range from mild, uncomplicated upper respiratory tract illness to severe lower respiratory tract infections, including pneumonia, bronchiolitis, croup, and exacerbations of asthma or wheezing. Most ARVIs are caused by a respiratory syncytial virus (RSV), influenza A and B, parainfluenza viruses types 1–4, adenoviruses, human metapneumovirus (hMPV), rhinoviruses, and coronaviruses [2,3]. Annual outbreaks of ARVI among children and the elderly, as well as people with immunodeficiencies, create a serious socioeconomic burden for all countries of the world, both developed and developing. To date, there are no available and effective specific drugs or vaccines that can significantly reduce the incidence and serious consequences of infections for the majority of ARVI pathogens (except for influenza viruses).

Human adenoviruses (AdV) are most commonly associated with pediatric illnesses of the upper respiratory tract, including the common cold [4]. New AdV serotypes are being constantly discovered, and to date, there are at least 85 various AdV serotypes belonging to seven species (A–G) that have been identified worldwide [5,6]. Among them, the AdV serotypes 1–5, 7, 14, and 21 are considered the main causes of ARVIs [7,8]. The only AdV vaccine currently approved is a live oral enteric coated AdV type 4 and 7 vaccine, which is licensed for use only in US military subjects, while there is currently no vaccine approved for the general population. (reviewed in [8]). The development of vaccines against adenoviruses is difficult due to the genetic diversity of these viruses. Virus-neutralizing antibodies have weak cross-reactivity between AdV serotypes, and it is very difficult to design a cross-protective AdV vaccine capable of protecting against a broad range of AdV serotypes using classical virological approaches [7]. A possible solution is the targeted induction of the immune response to the AdV epitopes that are conserved between different viral serotypes. In this case, the most effective system for the delivery of AdV T-cell epitopes to the epithelial cells of the upper respiratory tract are viral vectors that are capable of correctly presenting the target epitopes to the immune system [9], particularly for attenuated influenza viruses, which are used worldwide in live attenuated influenza vaccines (LAIVs) [10]. Recently, the idea of designing a bivalent vaccine against AdV and influenza was explored by generating recombinant influenza viruses that carry the AdV hexon protein antigenic epitope sequence inserted either in the non-structural (NS1) protein gene of the PR8 virus [11] or in the NA gene of a genetically engineered cold-adapted influenza virus [12]. In these studies, the structural neutralizing epitopes of the AdV hexon protein were inserted into the influenza genome, which resulted in the induction of neutralizing the AdV-targeted antibody, which protected the mice against challenges with wild-type AdV3 and AdV7 viruses. 

Earlier, we developed T cell-based LAIV virus-vectored vaccines against the human respiratory syncytial virus; these vaccines were safe, immunogenic, and protected mice against influenza and RSV without causing inflammatory disease [13,14]. Following this, we designed two LAIV-AdV T cell-based vaccine candidates by inserting conserved AdV T-cell epitopes into the open reading frame of full-length NA and truncated the NS1 proteins of H7N9 LAIV virus. We then tested the bivalent vaccines for their efficacy against influenza and human AdV5 in a mouse model. Importantly, a comprehensive analysis of the available data for the experimental CD4 and CD8 AdV epitopes was performed to select the most conserved and immunodominant epitopes to insert into the LAIV genome.

## 2. Materials and Methods

### 2.1. Viruses and Peptides

#### 2.1.1. Viruses

An H7N9 LAIV reassortant virus, generated earlier via reverse genetics [15], was used as a viral vector in this study. The reassortant influenza virus A/Shanghai/2/2013(H7N9)-PR8-IDCDC-RG32A (SH/13) bearing HA and NA from A/Shanghai/2/2013 (H7N9) and the remaining six genes from egg-adapted donor virus A/PR/8/34 (H1N1) were obtained from the Center for Disease Control and Prevention (CDC, Atlanta, GA, USA). Influenza viruses were propagated in 10–11 day-old chicken embryos and stored at −70 °C in aliquots. The infectious virus titer was determined in eggs by the method of Reed and Muench and expressed as log_10_EID_50_/mL.

The human serotype 5 adenovirus (AdV5) was obtained from the repository of human respiratory viruses of the Smorodintsev Research Institute of Influenza (Saint Petersburg, Russia). The adenoviruses were cultured in a Hep-2 cell culture (ATCC) grown in Dulbecco’s Modified Eagle Medium (DMEM), supplemented with a 5% FBS and 1× antibiotic-antimycotic (Gibco) solution at 37 °C in a 5% CO_2_ atmosphere. After the cytopathic effect (CPE) reached 95%–100% (usually on days 4–6 of incubation), the virus solution was harvested, clarified at 3500 rpm at 4 °C for 15 minutes, and stored in aliquots at −70 °C. The AdV titer was determined by spectrophotometry and real-time PCR analyses of a CsCl gradient-purified adenovirus. The AdV stocks were purified as described in [16]. The AdV genome titer was determined as described in [17] by measuring the OD at 260 nm of the purified virus, where one OD_260_ unit corresponded to 1.24 × 10^12^ AdV5 genome copies. The AdV DNA was extracted using a QIAamp DNA Mini Kit (Qiagen, Valencia, CA, USA). The real-time PCR reaction was set up by mixing 5 μL of a qPCRmix-HS SYBR ready-to-use mix (Evrogen, Moscow, Russia), 1 μl of forward primers (YAACCCCTTYTCKGGACCTC), and 1 μl of reverse (CAGTTGCTCTGCCTCTCCA) primers [18] (20 pmol each), as well as 3 μl of the DNA template and 15 μl dH_2_O. The PCR was conducted using a QuantStudio^TM^ 1 System (Thermo, Scientific, Rockford, IL, USA) programmed for one cycle of 5 min at 95 °C, followed by 40 cycles of 15 s at 94 °C, 15 s at 55 °C, and 30 s at 72 °C. The AdV genome copies were calculated from the standard curve by their Ct values using the QuantStudio^TM^ Design and Analysis Software v.1.5.1. The limit of detection of the assay was determined using serial virus dilutions and an AdV-negative specimen and corresponded to 10 DNA copies per µL.

#### 2.1.2. Peptides

The influenza Len/17-specific peptide (NP_366-374_ ASNENMD^TM^) was chemically synthesized by Almabion Ltd (Voronezh, Russia). The AdV5-specific peptide (DBP_418-425_ FALSNAEDL) was chemically synthesized by IQ chemicals Inc. (Saint Petersburg, Russia). The peptides had a purity of over 95%, as shown by high-performance liquid chromatography (HPLC). The peptides were reconstituted in dimethyl sulfoxide to a concentration of 1 mM and stored at −70 °C in single-use aliquots.

### 2.2. Selection of Adenovirus T-Cell Epitopes for Insertion into the LAIV Genome

The adenovirus protein sequences were obtained from the GenBank database; alignments were performed using the Unipro UGENE v1.29.0 software package. The search for promising conservative immunodominant AdV epitopes was carried out using the Immune Epitope Database (IEDB, http://www.iedb.org/), with a parallel analysis of the literature data (references to these studies are provided in Supplementary materials). We used data on experimentally confirmed epitopes deposited in the IEDB database, as well as a bioinformatic assessment of the content of the promising immunogenic epitopes in individual sequence fragments using IEDB. We evaluated the processing of the selected regions and the ability of individual peptides to bind to the human major histocompatibility complex (MHC), as well as to the MHC molecules of the mouse species, which will be used as a model for the experimental assessment of the vaccine’s immunogenicity and protective potential. The alignment of experimental epitopes found in IEDB on the selected AdV fragments was visualized using the Geneious 10.2.5 Software.

### 2.3. Rescue of the Recombinant LAIV Viruses

The recombinant influenza viruses, as well as the control LAIV reassortant strain, were rescued in the Vero cell via a Neon electroporation system (Invitrogen, Carlsbad, CA, USA) using a reverse genetics system developed for a cold-adapted master donor virus A/Leningrad/134/17/57 (H2N2) [19]. All viruses were fully sequenced using ABI BigDye Terminator v3.1 Cycle Sequencing kits and a capillary-based 3130xl Series Genetic Analyzer (Applied Biosystems, Foster City, CA, USA). Viruses were grown in 10 day-old embryonated chicken eggs at 33 °C. The allantoic fluid was harvested, clarified by centrifugation, and stored at −70 °C for further analyses.

### 2.4. In Vitro Studies

The temperature-sensitive (*ts*) and cold-adapted (*ca*) phenotypes of the rescued viruses were determined by titration at different temperatures in eggs: 38 °C compared to 33 °C for the *ts* phenotype, and 26 °C compared to 33 °C for the *ca* phenotype. The eggs were inoculated with 10-fold virus dilutions and incubated for either 48 hours (at 33 °C or 38 °C) or 6 days (at 26 °C). The virus titers in the eggs were calculated by the Reed and Muench method and expressed in terms of the log_10_ 50% egg infective dose (EID_50_)/mL.

### 2.5. Mouse Immunization and Protection Studies

#### 2.5.1. Ethical Statement

The animal studies were performed in accordance with the European Convention for the Protection of Vertebrate Animals used for Experimental and other Scientific Purposes (Strasbourg, France, 18.3.1986) and with the approval of the Institute of Experimental Medicine Ethics Committee (ethical approval number #3/19 dated 08 February 2019). Female 6–8 weeks old C57BL/6J mice were purchased from the Stolbovaya breeding laboratory and nursery of the Scientific Center for Biomedical Technologies of the Federal Medical and Biological Agency (Moscow Region, Moscow, Russia).

#### 2.5.2. Mouse Immunization and LAIV Virus Replication in the Mouse Respiratory Tract

**Figure 1:**
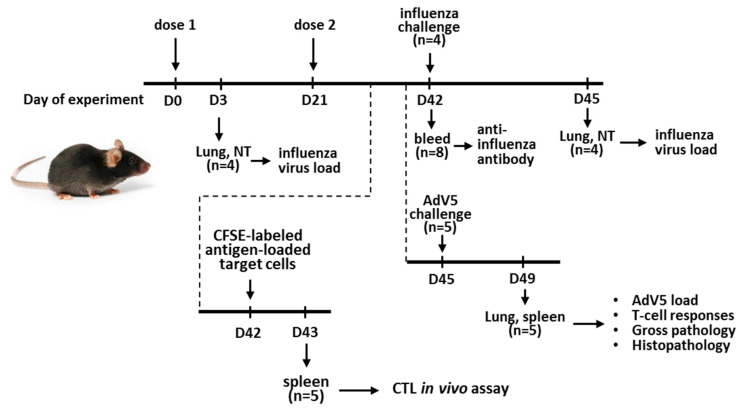
Overview of study design and procedures. Vaccine viruses were administered intranasally two times with a three-week interval. Influenza and AdV5 challenge viruses were also administered intranasally. Carboxyfluorescein succinimidyl ester (CFSE)-labeled peptide-pulsed splenic cells were administered intravenously via retro-orbital injection.

Groups of 8 to 10-week-old mice were immunized intranasally with LAIV strains at a dose of 10^6^ log_10_EID_50_ in a volume of 50 μL, twice at 21-day intervals. Mice in the placebo group received phosphate-buffered saline (PBS) in the same regimen (Figure 1). Replication of the vaccine viruses in the respiratory tracts of the mice was assessed on day 3 after the first immunization. Nasal turbinates and lungs were collected from four animals in each group and stored at −70 °C until being used for homogenization. Tissue homogenates were prepared in 1 mL of cold PBS supplemented with antibiotic-antimycotic using a TissueLyser LT small bead mill (QUIGEN, Germany). Supernatants collected after low-speed centrifugation were used to determine the viral titers by limiting their dilutions in eggs at a 33 °C incubation temperature.

#### 2.5.3. Protection against the Influenza Virus

Three weeks after the second vaccine dose, four mice in each group were challenged intranasally (i.n.) with the SH/13 influenza virus at a dose of 5.5 log_10_EID_50_ (corresponding to 100 MLD_50_) in a volume of 50 µL. Three days after the influenza challenge, the mice were sacrificed, and their lung and nasal turbinate tissues were collected to determine the viral loads, as described above (Figure 1).

#### 2.5.4. Protection against the Adenovirus

Three to four weeks after the second LAIV dose, five immunized mice were infected intranasally with 0.1 mL of wild type AdV5 virus diluted to contain 10^9^ genome copies per mL, as described in [20]. Four days after the AdV5 challenge, the mice’s lungs were collected for virological and pathomorphological studies. In addition, their spleens were collected at this time to assess the cell-mediated immune responses (Figure 1). Lung tissues from the AdV5-infected mice were homogenized in 1 mL sterile PBS using a TissueLyser LT small bead mill clarified by low-speed centrifugation. The AdV5 titers in the supernatants were then determined by real-time PCR, as described above.

### 2.6. Assessment of Immune Responses to the Recombinant Vaccines

#### 2.6.1. Antibody Immune Responses

Influenza-specific serum antibody titers were determined by a hemagglutination-inhibition assay (HAI), and the IgG was determined by an enzyme-linked immunosorbent assay (ELISA), as described previously [13]. For HAI, the serum samples were treated with chicken red blood cells to remove nonspecific inhibitors and quantified against four HA units of the H7N9 LAIV. The ELISA was performed with the same antigen as described earlier [13], except that ELISA plates were coated with 100 μL of sucrose-purified H7N9 LAIV virus antigen containing 16 HA units, and that serum samples were diluted three-fold starting from 1:40. The antibody levels were presented as the mean optical density (OD_450_) values for each serum dilution. In addition, the area under the curve (AUC) of the OD_450_ values was calculated for each serum sample using the trapezoidal rule.

#### 2.6.2. Cell-Mediated Immunity

Cell-mediated immunity was assessed using two assays: intracellular cytokine staining (ICS) and a CTL in vivo assay. T-cell-mediated immune responses were analyzed by ICS using gamma-interferon (IFNγ), as described in [14]. Briefly, murine splenocytes were isolated from five mice 4 days after the AdV5 challenge, and red blood cells were lysed by an ammonium-chloride potassium lysing buffer. For in vitro stimulation, the 1 × 10^6^ cells were incubated with an AdV DBP_418_ peptide at 1 µg/well for one hour in 100 µL of RPMI-1640 medium in 96-well microtitration U-bottom well plates. Then, 50 µL of RPMI-1640 medium supplemented with 30% FBS was added to a final FBS concentration of 10%. After 16–18 h, 50 µL GolgiPlug solution at a 1:250 dilution (Becton Dickinson, Franklin Lakes, NJ, USA), alone or with phorbol myristate acetate (PMA) as a positive control, was added, and the mixture was incubated for another five hours. After that, the samples were stained with ZombieAqua fixable viability dye (Biolegend, San Diego, CA, USA), PerCP-Cy5.5 anti-CD4 (100540, Biolegend), APC-Cy7 anti-CD8 (100714 Biolegend), PE anti-CD44 (103008 Biolegend), and BV-421 anti-CD62L (104435 Biolegend) antibodies over 20 minutes in the dark cold place. After this procedure, the samples were washed twice with 200 µL of a staining buffer. ICS was performed with a Cytofix/Cytoperm kit (Becton Dickinson) according to the manufacturer’s instructions, followed by staining the samples with an anti-IFNγ FITC-conjugated antibody (505806 Biolegend) over 20 minutes in a dark cold place. Then, the samples were washed twice with 200 µL of a wash buffer. The samples were fixed in 1% paraformaldehyde and stored in a dark cool place prior to the flow cytometric analysis. At least 100,000 events were measured using a Navios flow cytometer (Beckman Coulter, Brea, CA, USA). The data were analyzed using the FlowJo software (TriStar Inc., El Segundo, CA, USA); the percentage of peptide-specific T cells was calculated by subtracting the negative control from the cytokine-positive T cells. The gating strategy for the ICS assay is shown in Figure S1.

#### 2.6.3. CTL in vivo Assay

An in vivo cytotoxicity assay was performed on immunized mice three weeks after the second immunization, as described earlier [14]. Briefly, the target cells were prepared from splenocytes of naïve C57BL/6J mice (1 × 10^8^ splenocytes in 10 mL complete DMEM supplemented with 10% FBS) and loaded with 200 µg AdV DBP_418_ peptide (FALSNAEDL), Len/17-specific NP_366_ peptide (ASNENMD^TM^), or an equal volume of peptide diluent (PBS) for one hour at 37 °C with occasional mixing. Next, the cells were washed in PBS and stained with different concentrations of carboxyfluorescein succinimidyl ester (CFSE) in the PBS (60, 15, or 3.75 mM CFSE for the DBP_418_, NP_366,_ and PBS controls, respectively). The cells were then washed, resuspended in a Hanks solution, and mixed in equal amounts to a final concentration of 1 × 10^8^ cells/mL. Ten million prefiltered target cells were administered in 100 µL to anesthetized mice intravenously (i.v.) by retro-orbital injection (*n* = 5–6). The mixture of CFSE-labeled cells was administered to all mice at the same time to ensure that the proportions of the peptide-loaded and control target cells are identical. After 16–18 hours, the mice were sacrificed; the splenocytes were harvested and assessed by flow cytometry. At least 1 500 CFSE-positive events were measured using a Navios flow cytometer (Beckman Coulter). The ratio of peptide-loaded to control target cells was calculated as a normalized measure of the protein-specific cytotoxicity between the immunization groups. The gating strategy for the CTL in vivo assay is shown in Figure S2.

### 2.7. Histopathological Studies

The lungs of the mice (*n* = 5) were perfused in situ with 1 mL sterile PBS solution to remove blood from the vessels. The extracted lungs were then assessed for gross pathology, and virus-associated damage was assessed by the % of the affected tissue. A superior lobe of the right lung was fixed in 10% neutral buffered formalin for 48 hours. Then, paraffin-embedded tissues were cut into 5 μm thick sections on a microtome. Sections were deparaffinized and stained with hematoxylin and eosin (H&E), as well as with Alcian blue and safranin O (Kreiberg stain) to detect mucus and keratin, respectively [21,22]. The sections were examined using a Leica DM1000 microscope; micrographs at magnifications of 200–400× were obtained using an ADFPro20 camera. The adenovirus-related histopathological changes were assessed by the degree of bronchiolar epithelial cell damage, as well as the extent of alveolar, perivascular, and peribronchial infiltrations. The pathological outcomes were scored semiquantitatively for pathological changes on a scale of 0–4 [23,24] by assessing at least 20 fields in an individual mouse lung by a blinded pathologist using a light microscope. The sum of the pathological scores was calculated from all the parameters for each animal.

### 2.8. Statistical Analyses

Data were analyzed using the GraphPad Prism 6.0 software (GraphPad Software Inc., San Diego, CA, USA). The statistical significance of virological and immunological outcomes was determined by a one-way or two-way ANOVA followed by Tukey’s or Dunnett’s multiple comparison tests. The statistical significance of the LAIV viral titers in the eggs and mouse tissues was determined by a multiple t-test with a Holm–Sidak correction.

## 3. Results

### 3.1. Selection of Adenoviral T-Cell Epitopes for Designing the Recombinant LAIV-AdV Vaccines

The most common human respiratory adenoviruses belong to species C (serotypes 1, 2, and 5), B (3, 7, 11, 14, and 21), and E (4) [7]. Due to the significant variations in B-cell epitopes between different AdV serotypes [25], we aimed at designing recombinant LAIV viruses carrying several experimentally described human CD4 and CD8 T-cell epitopes conserved among different AdV serotypes. In addition, to assess the immunogenicity and protective potential of the chimeric vaccine viruses in BALB/c and C57BL/6J mouse models, we included immunodominant H2-d- and H2-b-restricted CD8 T-cell epitopes in the AdV cassette. 

**Figure 2:**
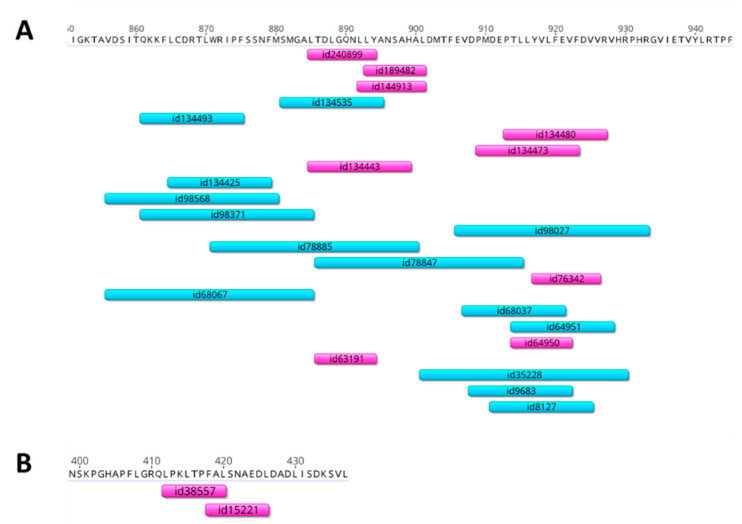
Distribution of the major histocompatibility complex (MHC) class I (purple) and class II (blue) restricted epitopes within the selected AdV cassette. (**A**) Distribution of human epitopes over the AdV5 hexon fragment (residues 855–935). (**B**) Distribution of mouse epitopes over the DBP fragment (residues 400–438). The epitope dataset was queried from the Immune Epitope DataBase in February 2020. The alignment was visualized using the Geneious 10.2.5 Software.

The whole-genome size of *Mastadenovirus* is 34–36 kbp [26]; sixteen genes are genus-common and have homologs in all genera of the *Adenoviridae* family, among which are polymerase, DNA-binding protein (DBP), and hexon, penton, and fiber proteins. Genome organization varies between species, and there are genus-specific genes and proteins. In addition, RNA splicing is a common feature for the adenovirus translation process [26]. Therefore, to find the promising immunogenic epitopes conserved among different AdV types/serotypes, we searched for literature data on experimentally established epitopes with positive reactions in a number of T-cell assays. Overall, the IEDB database contains 358 adenovirus epitopes located in hexon, penton, and fiber proteins with positive assay results; 350 of these proteins were detected in the human adenovirus species. The experimentally studied epitopes for the *Mastadenovirus* B, C, and E groups viruses are listed in Table S1. The most commonly studied is *Mastadenovirus* C, and the greatest number of epitopes was detected in the hexon protein. This protein contains multiple conserved regions enriched with immunogenic T-cell epitopes, which are conserved among different AdV groups [27,28,29,30]. Therefore, we selected the 80 amino acid-long C-terminus domain of the AdV hexon protein (residues 855 to 935) as an AdV cassette vaccine insert due to its high level of conservancy among different AdV serotypes (Figure S3) and also because it is enriched with experimental human T-cell epitopes (Figure 2A). This fragment contains a number of CTL epitopes conserved among various AdV serotypes, such as 63191 [29,31,32,33,34,35,36], overlapping epitopes 189482 [37], and 144913 [36,38], as well as HLA-II-restricted epitope 8127, the immune response to which was observed by ELISPOT in 78% of adults [39]. The full annotation of the experimental AdV T-cell epitopes located in the selected hexon fragment is provided in Table S2. In addition to the hexon fragment, we included in the cassette a fragment of the DNA-binding protein (DBP), which contains two overlapping H2-b and H2-d-restricted CTL epitopes (IEDB 38557 and 15221) (Figure 2B). These epitopes were described in a number of studies that demonstrated their immunodominance in mice [40,41,42,43]. These epitopes were also studied together with influenza-specific T-cell epitopes [44]. Importantly, this fragment only features confirmed T-cell epitopes recognized by mouse H2-b and H2-d MHC I alleles, with no epitopes described for humans. Therefore, the insertion of these epitopes in the AdV cassette will allow the assessment of the LAIV-AdV vaccine candidates in mouse models without safety concerns for humans. Notably, the inserted mouse epitope was not conserved among other human adenoviruses (Figure S4). Therefore, protection studies of the developed vaccines on the mouse model were restricted to the AdV5 serotype only.

### 3.2. Generation of Recombinant LAIV-AdV Viruses

**Figure 3:**
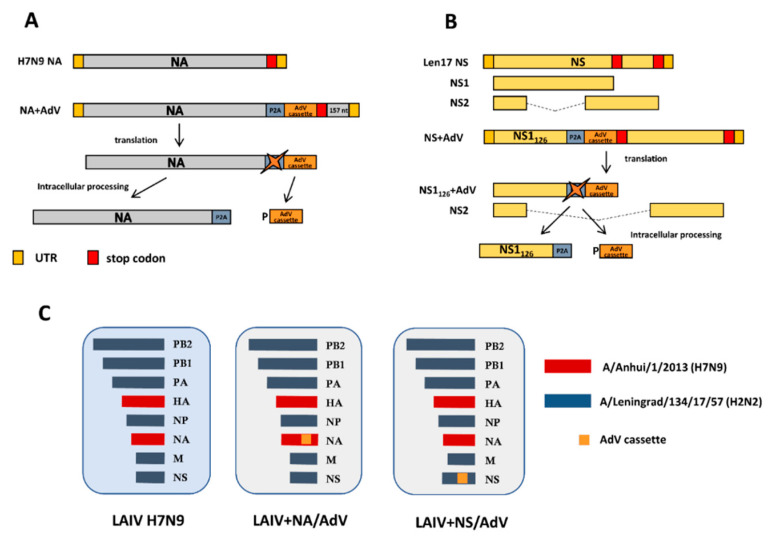
Overview of the designed recombinant influenza viruses. (**A**)**.** Scheme for generating the chimeric influenza NA gene carrying AdV cassette. (**B**)**.** Scheme for generating the chimeric influenza NS1 gene-carrying AdV cassette. (**C**)**.** Genome composition of the live attenuated influenza vaccine (LAIV) viruses. P2A: porcine teschovirus-1 P2A self-cleavage site (GSGATNFSLLKQAGDVEENPG↓P).

The selected AdV fragments enriched with T-cell epitopes were combined into a cassette with the N-terminus leading sequence of the P2A self-cleaving peptide (Figure 3). We used a previously described strategy for generating the prototype LAIV-AdV vaccine candidates [14]. The AdV construct was inserted into the C-terminus of the influenza H7N9 NA protein so that the full-length NA protein was translated and incorporated into the viral particle, whereas the cleaved AdV cassette remained inside the infected cell (Figure 3A). Another recombinant LAIV-AdV candidate was designed to produce a C-terminally truncated NS1 protein that retained N-terminal 126 amino acids, followed by the P2A self-cleavage site and the AdV cassette (Figure 3B). This C-terminal truncation was selected based on the previous studies for the successful generation of attenuated and immunogenic influenza viruses [45,46]. The two recombinant LAIV viruses (LAIV+NA/AdV and LAIV + NS/AdV, Figure 3C) were rescued in certified Vero cells and fully sequenced, and their genomic identities were confirmed. Furthermore, the genetic stability of the rescued viruses was assessed by 10 serial virus passages in eggs, followed by full-genome sequencing. The AdV inserts were maintained throughout these passaging procedures, and no spontaneous mutations were observed (data not shown).

### 3.3. Assessment of LAIV-AdV Virus Replication in vitro and in vivo

**Figure 4:**
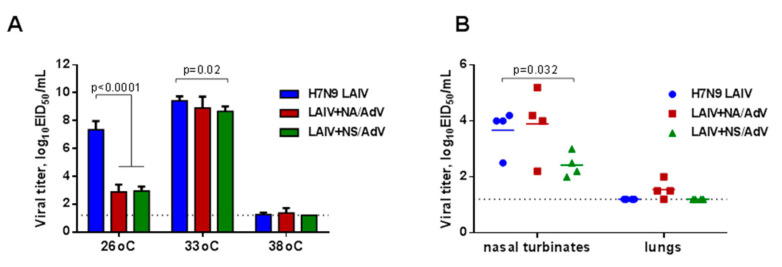
Replication of the LAIV viruses in vitro and in vivo. (**A**)**.** Infectious viral titers in eggs at different incubation temperatures. (**B**)**.** LAIV viral titers in mouse respiratory tissues at day 3 after intranasal administration of the tested viruses at a dose of 10^6^ EID_50_. The dotted line indicates the limits of virus detection. The statistical significance of the differences in the viral titers between the test groups was determined by a Holm–Sidak multiple t-test.

The replicative characteristics of the newly generated recombinant LAIV-AdV viruses were first assessed in eggs at different temperatures, with H7N9 LAIV as a viral vector control. Both LAIV+AdV variants replicated to slightly lower titers in eggs at an optimal temperature of 33 °C than the control virus, but only the LAIV+NS/AdV virus reached statistical significance (Figure 4A). Importantly, the LAIV-AdV recombinant viruses were highly temperature-sensitive, which is a necessary feature of attenuated influenza viruses; however, the cold-adapted phenotype, also typical for LAIV viruses, was impaired in both recombinant strains. Notably, a similar loss of the *ca* phenotype was observed for other LAIV-based vectored vaccines carrying T-cell epitopes of the respiratory syncytial virus [13]. Nevertheless, both LAIV-AdV vaccine candidates were able to replicate in the upper respiratory tract of C57BL/6J mice (Figure 4B). LAIV+NS/AdV had significantly lower titers in their nasal turbinates than the H7N9 LAIV virus, which is consistent with previous studies that observed that the truncation of the NS1 protein up to 126 amino acids reduced the virus titer in mouse tissues by 100 times [47,48]. Most importantly, neither the LAIV virus tested in this study could actively replicate in the lungs, indicating the attenuated phenotype of the viruses.

### 3.4. Anti-influenza Antibody Immune Responses and Protection

**Figure 5:**
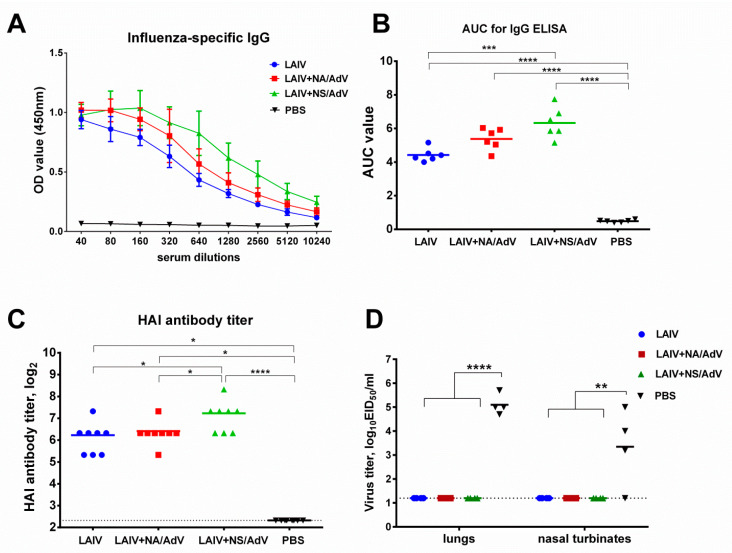
Immunogenicity and protection against influenza virus. (**A**) Serum influenza-specific IgG measured by ELISA using the whole H7N9 LAIV virus as an antigen. The OD450 values are shown for the indicated serum dilutions (*n* = 6). (**B**) The area under the curve values (AUC) for the serum IgG ELISA were calculated for each mouse using the trapezoidal rule. (**C**) Hemagglutination inhibition titers to the H7N9 LAIV virus (*n* = 8). (**D**) Tissues were collected on day 3 after infection with the H7N9-PR8 influenza virus (*n* = 4). Representative data from two independent experiments are shown. Data were compared using a one-way ANOVA followed by Tukey’s multiple comparison test. The asterisks refer to the level of significance: * *p* < 0.05, ** *p* < 0.01; *** *p* < 0.001, **** *p* < 0.0001. Dotted lines indicate the limits of viral detection.

Since the recombinant LAIV-AdV viruses are intended for use as a bivalent vaccine, it was important to determine whether the insertion of a foreign antigen can impair anti-influenza immunity and protective potential. A two-dose immunization with the chimeric viruses resulted in the induction of high antibody titers to the H7N9 LAIV virus, in both the HAI and ELISA assays (Figure 5). Surprisingly, the LAIV+NS/AdV virus was found to be significantly more immunogenic than both the LAIV vector and the LAIV+NA/AdV vaccines, both in ELISA (Figure 5A,B) and HAI (Figure 5C), despite the reduced replication in nasal turbinates observed at day 3 after immunization (Figure 5B). Nevertheless, all three vaccines fully protected immunized mice against replications in the respiratory tract of the homologous challenge virus administered at a high dose (100 MLD_50_), whereas this challenge virus reproduced to high titer levels in mock-immunized mice, both in the lungs and the nasal turbinates (Figure 5C). Although we did not measure mucosal IgA antibody levels in immunized mice, our previous results indicate that this arm of adaptive immune responses may also contribute to the protection [49]. Overall, these data suggest that the inserted immunogenic epitopes of AdV did not interfere with influenza-specific antibody immune responses induced by LAIV intranasal immunization.

### 3.5. Functional Cytotoxic T-Cell Responses to Influenza and Adenovirus CTL Epitopes

**Figure 6:**
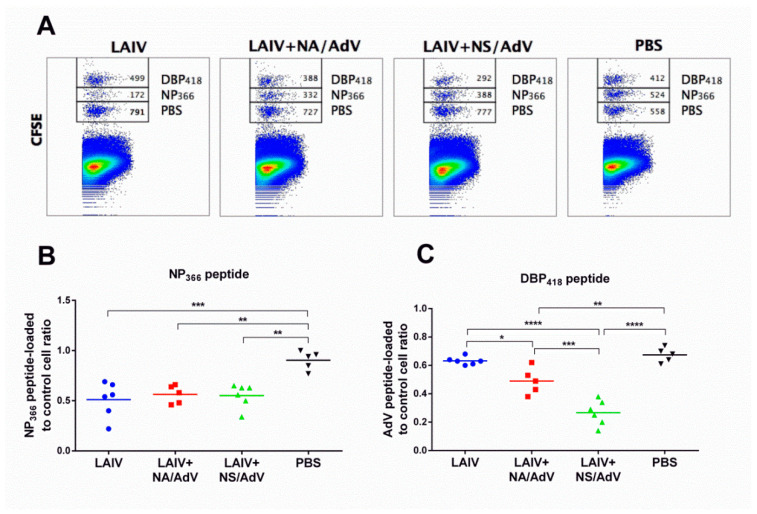
Assessment of the functional activity of the induced cytotoxic T-cell response in a CTL in vivo assay (*n* = 5–6). (**A**) Representative flow cytometry plots for the mouse splenocytes collected 18 hours after intravenous (i.v.) injection of a mixture of target cells loaded with NP_366_, DBP_418,_ or PBS. The number of surviving target cells for each CFSE concentration out of 1 500 CFSE-positive events is shown. (**B**) Ratio of NP_366_ peptides loaded to the control cells for all study groups. (**C**) Ratio of the DBP_418_ peptide loaded to the control cells for all study groups. Data were compared using a one-way ANOVA followed by Tukey’s multiple comparison test. The asterisks refer to the level of significance: * *p* < 0.05, ** *p* < 0.01; *** *p* < 0.001, **** *p* < 0.0001.

LAIVs are capable of inducing functional cytotoxic T-cell responses in mouse models; therefore, we assessed the ability of the CTLs generated in response to LAIV-AdV immunization to kill target cells loaded with peptides corresponding to the influenza immunodominant epitope (NP_366_) and the AdV immunodominant epitope (DBP_418_). A mixture of peptide-loaded and PBS-loaded splenocytes (each population labeled with a unique concentration of CFSE) was administered intravenously to the immunized and control mice, and the number of splenic cells labeled with each CFSE concentration was assessed 18 hours later by flow cytometry (Figure 6A). The killing activity of the cytotoxic T cells induced by all three vaccine viruses was equal to that of the NP_366_-loaded target cells as the concentrations of these cells were significantly reduced in the vaccinated mice compared to the mock-immunized animals, with no differences observable between the vaccines (Figure 6B). The CTL in vivo assay confirmed that the DBP_418_ peptide of the human adenovirus (FALSNAEDL) is immunogenic and can induce functional cytotoxic CD8 T cells, which can efficiently kill the peptide-loaded cells in the immune mice (Figure 6C). Strikingly, the LAIV+NS/AdV recombinant virus was significantly more immunogenic against the AdV peptide than its corresponding LAIV+NA/RSV counterpart. As these two recombinant viruses were equally immunogenic with regards to the influenza virus antigen, the superior immunogenicity of the AdV+NS/AdV candidate to the transgene suggests that the expression of the AdV cassette within the truncated NS1 open reading frame can be more advantageous for stimulating T-cell responses to foreign inserts than expression within the NA protein of the influenza virus vector.

### 3.6. Protection against Wild-Type AdV5 Infection

**Figure 7:**
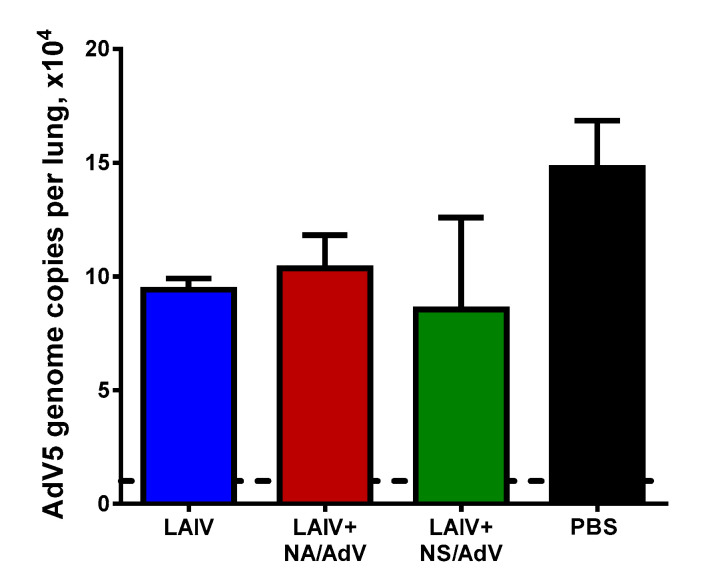
Detection of AdV5 genome copies in the lungs of immunized mice post-AdV5 challenge. The mouse lungs (*n* = 5) were collected four days after AdV5 infection, and AdV5 titers were determined by real-time PCR. The dashed line indicates the detection limit of the assay. The data were compared using a one-way ANOVA followed by Tukey’s multiple comparison test.

To determine if the induced CTL immunity to the AdV epitope can protect mice against AdV5-induced pathology, we infected the immunized mice i.n. with the AdV5 virus at a dose of 10^8^ virus particles and assessed the protection on day 4 after infection. It is known that type 5 human adenovirus can cause severe pulmonary pathology in C57BL/6 mice, although the virus cannot produce infectious progeny in mouse lungs [20]. Indeed, low AdV5 genome copy numbers were detected in the mouse lungs four days after infection, and there were no significant differences between the test groups (Figure 7). These data indicate that the AdV-specific cell-mediated immunity was not sufficient to facilitate viral clearance from the mouse lungs. 

**Figure 8:**
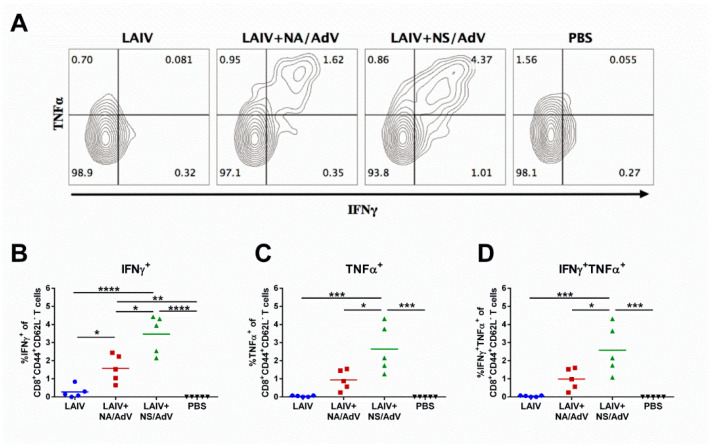
Recall AdV-specific CD8+ effector memory immune responses in mouse splenocytes four days after the AdV5 challenge. Splenocytes were stimulated with the AdV peptide FALSNAEDL, and the levels of IFNγ and/or TNFα-producing cells were assessed by ICS. (**A**) Representative flow cytometry plots for the CD8 + CD44 + CD62L- T-cell subsets. (**B**) Levels of IFNγ-producing Tem cells. (**C**) Levels of TNFα-producing Tem cells. (**D**) Levels of double-positive cytokine-producing Tem cells. Representative data from two independent experiments are shown. Data were compared with a one-way ANOVA followed by Tukey’s multiple comparison test. The asterisks refer to the level of significance: * *p* < 0.05, ** *p* < 0.01, *** *p* < 0.001, **** *p* < 0.0001.

We further assessed the magnitude of recall CD8 T-cell responses in immunized mice upon the AdV5 challenge. Both LAIV-AdV candidates stimulated effector memory T-cell responses, which were recalled shortly after the immunized mice encountered a natural AdV5 infection (Figure 8). Similar to the CTL in vivo results, the LAIV + NS/AdV recombinant virus was significantly more immunogenic than its LAIV+NA/AdV counterpart and resulted in a higher magnitude of recall CD8 Tem responses. Interestingly, the majority of LAIV + NS/AdV vaccine-induced effector memory T cells were characterized by dual cytokine production (IFNγ + TNFα), whereas a lower proportion of the IFNγ-positive Tem cells from the LAIV + NA/AdV test group were also able to secrete TNFα (Figure 8B–D). 

**Figure 9:**
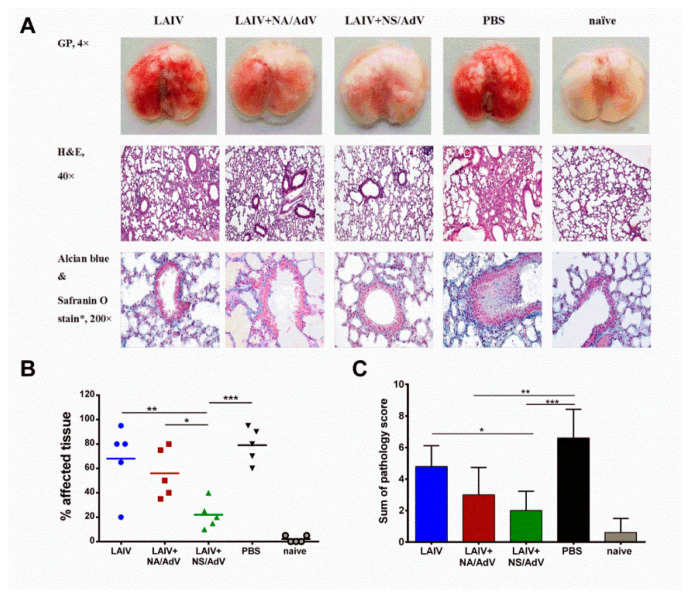
Histopathological evaluation of the lungs of the immunized mice 4 days after AdV5 infection. (**A**) Representative gross pathology (GP) and histopathology micrographs. H&E: hematoxylin and eosin stain. (**B**) During dissection, the lungs were examined macroscopically, and an estimate of the percentage of the affected tissue (a bright red color) was recorded. (**C**) Sum of pathology score is determined based on the degree of bronchiolar epithelial cell damage, as well as the extent of alveolar, perivascular, and peribronchial infiltrations. The data were compared using a one-way ANOVA followed by Tukey’s multiple comparison test. The asterisks refer to the level of significance: * *p* < 0.05, ** *p* < 0.01, and *** *p* < 0.001.

The detection of polyfunctional CD8 T cells in the LAIV+NS/AdV group after the AdV5 challenge indicates that this vaccine candidate induced robust protective CD8 memory T-cells with strong antiviral responses through the expression of antiviral effector cytokines upon encounter the antigen [50]. Indeed, mice from this group were almost fully protected against the pulmonary pathology caused by AdV5 infection, seen both macroscopically and microscopically (Figure 9). In contrast, the AdV5 infection caused significant lung pathology in the non-immunized mice observed at a microscopic level (Figure 9A), as well as histopathological changes, such as bronchiolar epithelial cell damage and alveolar and peribronchial inflammatory response, which is known to be most pronounced at days 4–5 post-infection [20,24]. Although the LAIV+NA/AdV-vaccinated mice had substantially reduced histopathological end scores in their lung tissues compared to the non-immunized mice (Figure 9C), their AdV-specific antiviral immunity was not sufficient to protect the animals against macroscopic pathological changes (Figure 9B). These results indicate the strong protective role of the multifunctional memory CD8 T-cell responses induced by the LAIV+NS/AdV recombinant virus in the protection against natural AdV5 infections.

## 4. Discussion

In this study, we developed two viral-vectored vaccines capable of protecting against influenza virus and human adenoviruses using an attenuated influenza virus as a vector delivery system. Unlike recently described recombinant influenza viruses carrying the AdV hexon immunogenic B-cell epitope [11,12], our constructs are based on a licensed LAIV backbone, which has been used for several decades and has a strong safety record in humans [51]. Furthermore, we were focused on the idea of augmenting the induction of T-cell immune responses to the antigen of interest, as it is well known that particular vaccine vectors can properly present foreign antigens to the immune system [52]. Cytotoxic T cells are recognized as major players in the immune response against viral infections because they kill infected cells, not the pathogen itself, and because they recognize epitopes that are conserved among different viral strains [53]. Another advantage of designing T cell-based vectored vaccines is that there is the possibility to significantly reduce the size of the antigen and leave only key immunodominant epitopes, whereas vaccines designed to induce neutralizing antibodies against the desired pathogen most often require large inserts of the foreign antigens [9]. Given that influenza viruses have a limited genome capacity for the insertion of foreign genetic information, the design of compact immunogens (immunodominant epitopes) to be incorporated into the viral genome is preferable. Here, we selected promising genomic fragments of human adenoviruses, which are compact but contain at least 24 conserved CD8 and CD4 T-cell epitopes, which were proven to be immunogenic for humans through a number of T-cell assays (see Table S2). 

Due to the lack of available humanized animal models, a proof of concept study has been undertaken. We incorporated two overlapping immunodominant mouse CD8 T-cell epitopes to evaluate the immunogenic and protective potential of the T cells developed after the intranasal delivery of these epitopes by the attenuated influenza viral vector. The finding that the human AdV epitopes deposited in the immune epitope database are dominated by the AdV type 5 virus is not surprising as AdV5 is the most frequently used serotype for AdV vector-based vaccine design and gene therapy [54]. For AdV-vectored vaccines, AdV-specific T-cell immunity is considered an undesirable effect of vaccination, and the researchers have attempted to overcome the host immunity to these vectors [55]. In contrast, here, we explored the opportunity to design an AdV vaccine using key experimental T-cell epitopes known to be immunogenic in humans. More importantly, the selected epitopes are cross-reactive among AdV serotypes and have the potential for broad protection against a range of adenoviruses. It was demonstrated that the immunodominance of AdV-derived CTL epitopes can interfere with the immunogenicity of the inserted transgene in AdV vector-based immunizations [43]. However, in our study, the insertion of the immunodominant AdV CD8 T-cell epitope did not affect the establishment of influenza virus-specific antibody and functional cytotoxic T-cell responses, regardless of the magnitude of the induced AdV-specific responses. These data suggest that recombinant LAIV viruses carrying immunogenic foreign epitopes induce balanced T-cell responses both for the insert and for the vector itself. However, more research is needed to elucidate the precise immunologic mechanisms underlying the development of cell-based immunity by LAIV-based vectored vaccines and if a pre-existing immunity to the influenza virus would interfere with such immune responses. The induced AdV peptide-specific CTL response demonstrates that the LAIV virus correctly presented the foreign T-cell epitope to the immune systems of the mice.

The selection of the AdV type 5 for our study was also based on the ability of this virus to induce pneumonia in animal models, such as cotton rats and C57BL/6 mice, thus providing an opportunity to assess the protective potential of the designed constructs [20]. This mouse model does not support the active replication of AdV5 in the lungs; however, the induced AdV-specific CD8 T cells could not significantly accelerate the clearance of the virus in our study. This could be due to the use of real-time PCR to detect viral DNA, which can detect a non-viable virus in cells attacked by AdV5-specific CD8 T cells. The limitation of our study is that we did not measure infectious AdV5 titers in mouse tissues, which could reveal the effect of AdV-specific T cells on the elimination of the virus from the respiratory tract. Nevertheless, to the best of our knowledge, for the first time, we demonstrated a clear relationship between the magnitude of induced AdV-specific CTL responses and protective effects against lung injury following AdV5 infection in C57BL/6J mice. The NS1-based LAIV-AdV vaccine was a more potent inducer of AdV-specific CTL responses than a similar vaccine with insertion in the NA gene. This could be due to the truncation of the NS1 protein, a known inhibitor of type I IFN production, and the induction of antiviral responses [56], which can result in enhanced influenza-specific immune responses [45,57]. Indeed, the influenza-specific hemagglutination inhibiting antibody, as well as virus-binding serum IgG antibody, were induced at a higher magnitude after vaccination with the LAIV+NS/AdV vaccine compared to the LAIV+NA/AdV and LAIV vector study groups. However, our CTL in vivo data show the identical killing activity of the induced NP_366_-specific T cells after immunization with NA- and NS-based AdV vaccines, whereas the killing activity of the AdV-specific CTLs was significantly different between the two vaccines, indicating that the magnitude of the CTL response to the foreign antigen is dependent on the level of its expression inside the infected cell. It is known that NS1 is expressed at a much higher level than the NA protein [58]. Noteworthy, here we demonstrated that vaccination with the recombinant viruses resulted in AdV-specific effector memory T-cell responses in spleen post-AdV5 challenge, whereas secondary lymphoid tissues usually have Tcm memory phenotype, rather than Tem phenotype [59]. However, it is known that natural influenza viral infection, but not immunization with inactivated vaccines, can lead to Tem responses in the mouse spleen [60]. In our study, the vaccine was administered intranasally, mimicking a natural infection, and the AdV-specific Tem responses were evaluated after infection with another respiratory viral infection. The presented data indirectly suggest that the human adenovirus T-cell epitopes inserted into the AdV cassette in our recombinant viruses will also be efficiently expressed within an infected cell and correctly presented by the immune system of the host.

## 5. Conclusions

Overall, in this study, we described new recombinant influenza viruses based on the LAIV virus backbone, which carries a number of immunogenic CD4 and CD8 T-cell epitopes of human adenoviruses. The insertion of an immunodominant H2^b^-restricted CD8 T-cell epitope from group C adenoviruses allowed us to demonstrate the robust protective potential of these vaccines against influenza and AdV5 infections in C57BL/6J mice. Further studies of immunogenicity and the ability to protect against a variety of AdV serotypes using humanized animal models are warranted. Furthermore, here, we explored two sites within the LAIV genome for the insertion of a transgene, thus providing new perspectives on generating multivalent vaccines against different respiratory viruses by inserting their promising immunogenic epitopes into the LAIV backbone.

## Supplementary Materials

The following are available online at https://www.mdpi.com/2076-393X/8/2/196/s1, Figure S1: Gating strategy for the assessment of effector memory CD8 T-cell responses in mice using intracellular cytokine staining assay, Figure S2: Gating strategy for CTL in vivo assay, Figure S3: Alignment of a C-terminus fragment of AdV hexon (residues 855-935) among various AdV serotypes belonging to different AdV groups, Figure S4: Alignment of a DBP region (residues 400-438) among various AdV serotypes belonging to different AdV groups, Table S1: Experimentally established T- and B-cell epitopes of Human Adenoviruses deposited in the Immune Epitope Database (IEDB), Table S2: Experimentally established T-cell epitopes located within human adenovirus hexon region 855-935.

## Acronym list and glossary

**AdV**	adenovirus
**ARVI**	acute respiratory viral infection
**ATCC**	American Type Culture Collection
**AUC**	area under the curve
**BSA**	bovine serum albumin
***Ca***	cold-adapted
**CFSE**	carboxyfluorescein succinimidyl ester dye
**CPE**	cytopathic effect
**Ct**	cycle of threshold
**CTL**	cytotoxic T lymphocyte
**DBP**	DNA-binding protein
**DMEM**	Dulbecco’s Modified Eagle Medium
**EID_50_**	50% egg infective dose
**ELISA**	enzyme-linked immunosorbent assay
**ELISPOT**	enzyme-linked immunospot assay
**FBS**	fetal bovine serum
**GP**	gross pathology
**hAdV**	human adenovirus
**HAI**	hemagglutination inhibition assay
**H&E**	hematoxylin and eosin
**HLA**	human leucocyte antigen
**hMPV**	human metapneumovirus
**HPLC**	high-performance liquid chromatography
**IEDB**	immune epitope database
**IFNγ**	interferon gamma
**ICS**	intracellular cytokine staining
**i.n.**	intranasal
**i.v.**	intravenous
**LAIV**	live attenuated influenza vaccine
**LD_50_**	50% mouse lethal dose
**Len/17**	A/Leningrad/134/17/57 (H2N2) virus
**MHC**	major histocompatibility complex
**NA**	neuraminidase
**NP**	nucleoprotein
**NS**	non-structural protein
**NT**	nasal turbinates
**OD**	optical density
**P2A**	self-cleaving site from porcine teschovirus-1
**PBS**	phosphate-buffered saline
**PCR**	polymerase chain reaction
**PMA**	phorbol myristate acetate
**PR8**	A/PR/8/34 (H1N1) virus
**RSV**	respiratory syncytial virus
**SH/13**	PR8-based reassortant virus bearing HA and NA from A/Shanghai/2/2013 (H7N9)
**Tcm**	central memory T cells
**Tem**	effector memory T cells
**TNFα**	tumor necrosis factor alpha
***Ts***	temperature sensitive
