# Supplementary material for: Recombinant Live Attenuated Influenza Vaccine Viruses Carrying Conserved T Cell Epitopes of Human Adenoviruses Induce Functional Cytotoxic T Cell Responses and Protect Mice against both Infections
Source: Vaccines (Basel). 2020 Apr 24;8(2):196. doi: 10.3390/vaccines8020196 (PMC7349758; doi:10.3390/vaccines8020196)
Supplement: Supplementary file 1 [file vaccines-08-00196-s001.pdf]

**Supplementary material** for paper by Isakova-Sivak et al. "**Recombinant live attenuated influenza vaccine viruses carrying conserved T-cell epitopes of human adenoviruses induce functional cytotoxic T-cell responses and protect mice against both infections**"

**Table S1.** Experimentally established T- and B-cell epitopes of Human Adenoviruses deposited in the Immune Epitope Database (IEDB)

| **Viral protein** | **Total 320** | T-cell epitopes | | | | | B-cell epitopes | | |
|---|---|---|---|---|---|---|---|---|---|
| | | Human | | Mouse | | | **B all** | **Human** | [illegible] |
| | | **MHC I** | **MHC II** | **H2-b** | **H2-d** | **MHC II** | | | |
| **Human mastadenovirus E** | 1 | | | | | | | | |
| Hexon | 1 | | | | | | **1** | | |
| **Human mastadenovirus B** | 69 | | | | | | | | |
| Fiber | 24 | | | | | | **24** | | [illegible] |
| Hexon | 43 | 12 | 0 | | | | 31 | | [illegible] |
| pVII | 2 | | 2 | | | | | | |
| **Human mastadenovirus C** | 251 | | | | | | | | |
| Hexon | 159 | 39 | 124 | | 1 | | 9 | 5 | [illegible] |
| Fiber | 40 | 1 | 2 | | | | 38 | | [illegible] |
| DNA polymerase | 3 | 1 | 2 | | | | | | |
| DNA-binding protein | 2 | | | 1 | 2 | | | | |
| E1B 55 kDa protein | 7 | | | 6 | | 1 | | | |
| E1B protein, small T-antigen | 2 | | 1 | | | | 1 | | |
| Early E1A protein | 10 | 1 | 2 | 7 | | | 4 | | |
| Hexon-interlacing protein | 2 | | 2 | | | | | | |
| I-leader protein | 1 | 1 | | | | | | | |
| 23K endopeptidase | 1 | | | | | | 1 | | [illegible] |
| Packaging protein 3 | 1 | | 1 | | | | | | |
| Penton protein | 12 | 8 | 5 | | | | 1 | | |
| Pre-core protein X | 1 | | | | | | 1 | | |
| Pre-early 3 receptor internalization and degradation alpha protein | 1 | | 1 | | | | | | |
| Pre-hexon-linking protein VIII | 3 | | 3 | | | | | | |
| Pre-histone-like nucleoprotein | 2 | | 1 | | | | 1 | | |
| Pre-protein VI | 2 | | 2 | | | | | | |
| Adenovirus death protein | 1 | | 1 | | | | | | |

**Table S2.** Experimentally established T-cell epitopes located within human adenovirus hexon region 855-935

| IEDB ID | Epitope sequence | Location | Serotype[1] | Cross-reactive | Allele | Ass |
|---|---|---|---|---|---|---|
| 68067 | VDSITQKKFLCDRTLWRIPFSSNFMSMGAL | 856-885 | hAdV 5 | C (B[4]) | HLA-DR17 HLA class II | IFN<br>pat<br>pro<br>cyt<br>deg<br>IL-<br>TN<br>IL- |
| 98568 | VDSITQKKFLCDRTLWRIPFSSNFM | 856-880 | hAdV 5 | C | HLA class II | IFN |
| 98371 | QKKFLCDRTLWRIPFSSNFMSMGAL | 861-885 | hAdV 5 | C | HLA class II | IFN |
| 134493 | QKKFLCDRTLWRIPF | 885-899 | hAdV 5 | C | HLA class II | IFN |
| 134425 | LCDRTLWRIPFSSNF | 865-879 | hAdV 5 | C | HLA class II | IFN |
| 78885 | WRIPFSSNFMSMGALTDLGQNLLYANSAHA | 871-900 | hAdV 5 | C | HLA class II | IFN |
| 134535 | SMGALTDLGQNLLYA | 881-895 | hAdV 5 | C | HLA class II | IFN |
| 134443 | LTDLGQNLLYANSAH | 885-899 | hAdV 5 | C | HLA-A*01:01 | IFN |
| 240899 | LTDLGQNLLY | 901-910 | hAdV 2 | C | HLA-A*01:01 | cyt<br>IFN<br>IL-<br>TN<br>act<br>qua |
| 63191 | TDLGQNLLY | 902-910 | hAdV 5 | C (B[4]) | HLA-A1 | cyt<br>IFN<br>qua<br>pro |
| 78847 | TDLGQNLLYANSAHALDMTFEVDPMDEPTL | 886-915 | hAdV 5 | C | HLA class II | IFN |
| 144913 | LLYANSAHAL | 892-901 | hAdV 5 | C | HLA-A2 HLA-A*02:01 | qua<br>IFN<br>IL-<br>per<br>TN<br>cyt<br>pro |
| 189482 | LYANSAHAL | 893-901 | hAdV 11 | all | HLA-A*24:02 | IFN |
| 35228 | LDMTFEVDPMDEPTLLYVLFEVFDVVRVHR | 901-930 | hAdV 5 | 5 | HLA class II | IFN |
| 8127 | DEPTLLYVLFEVFDV | 911-925 | hAdV 5 | C | HLA-DR HLA-DPw4 HLA-DPB1*04:01 HLA-DR7 | cyt<br>IFN<br>pro |

| | | | | | | |
|---|---|---|---|---|---|---|
| | | | | | HLA-DR15 | pat |
| 98027 | EVDPMDEPTLLYVLFEVFDVVRVHRPHR | 906-930 | hAdV 5 | 5 | HLA class II | IFN |
| 68037 | VDPMDEPTLLYVLFE | 907-921 | hAdV 5 | C | HLA-DR HLA class II | IFN<br>IL-<br>IL-<br>CC<br>CC<br>CC<br>CX<br>gra<br>IL-<br>IL-<br>onc<br>pro<br>TN<br>IL-<br>IL-<br>IL-<br>IL-<br>IL-<br>IL-<br>MI<br>per<br>TN |
| 9683 | DPMDEPTLLYVLFEV | 908-922 | hAdV 5 | C | HLA-DR | IFN |
| 134473 | PMDEPTLLYVLFEVF | 909-923 | hAdV 5 | C | HLA-DQB1*04:02 HLA-C*07:02 HLA-DQB1*04:02 HLA-C*07:02 HLA-DQB1*04:02 HLA-C*07:02 | IFN<br>cyt |
| 134480 | PTLLYVLFEVFDVVR | 913-927 | hAdV 5 | C | | IFN<br>cyt |
| 64950 | TLLYVLFEV | 914-922 | hAdV 5 | C | HLA-A*02:01 HLA-DPB1*04:01 HLA-DR | IFN<br>qua<br>cyt<br>deg<br>pro |
| 64951 | TLLYVLFEVFDVVRV | 914-928 | hAdV 5 | C | HLA-DR | IFN |
| 76342 | YVLFEVFDVV | 917-926 | hAdV 5 | C | HLA-A*02:01 | IFN<br>qua |

| | | | | | | |
|---|---|---|---|---|---|---|
| 78743 | LYVLFEVFDVVRVHRPHRGVIETVYLRTPF | 916-945 | hAdV 5 | 5 | HLA class II | IFN |

[1] – AdV serotype used in the original study;
[2] – T-cell assays used in these studies;
[3] – according to the IEDB data;
[4] – cross-reactivity is shown based on sequence identity; experimental confirmation for cross-reactivity with this serotype is shown in parenthesis.

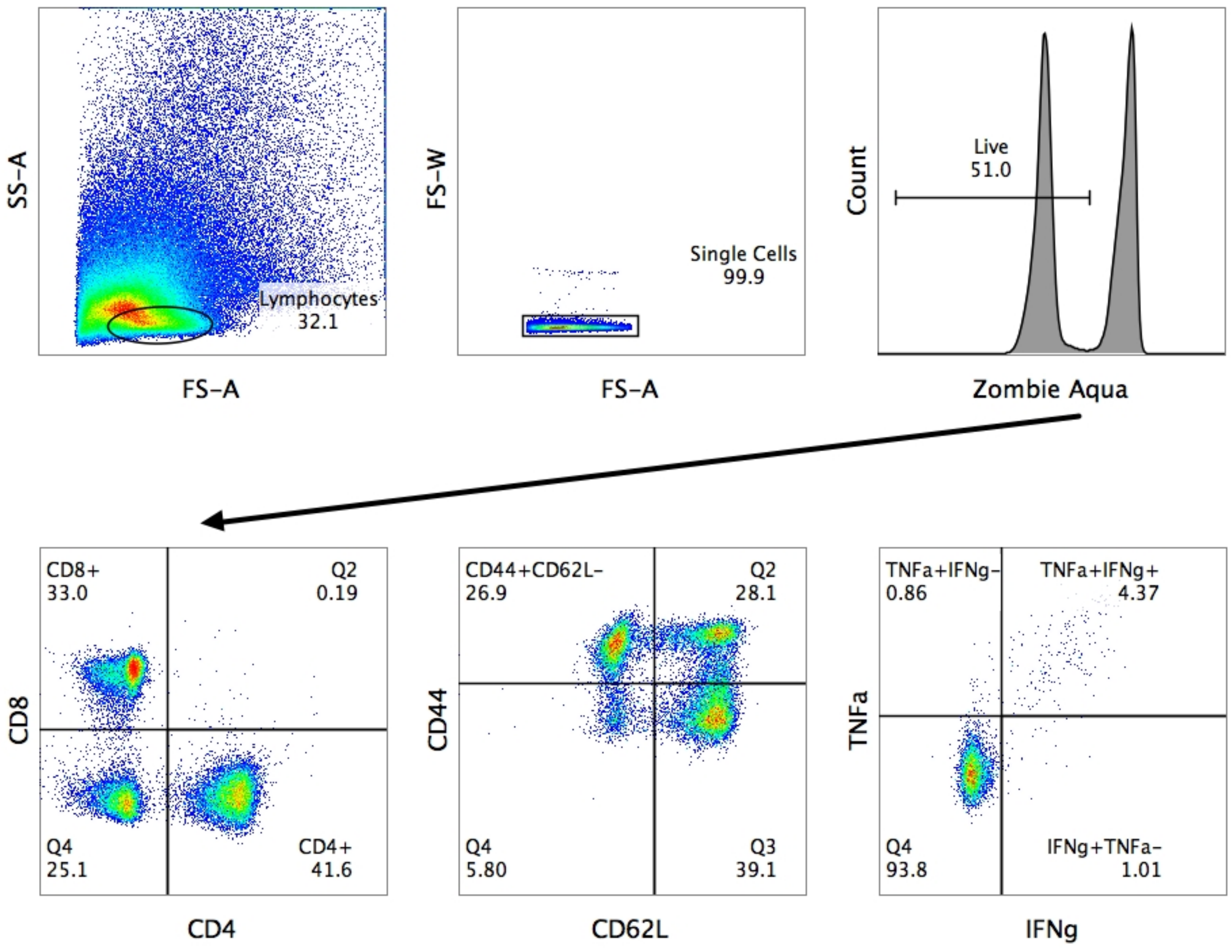


**Figure S1.** Gating strategy for the assessment of effector memory CD8 T-cell responses in mice using intracellular cytokine staining assay.

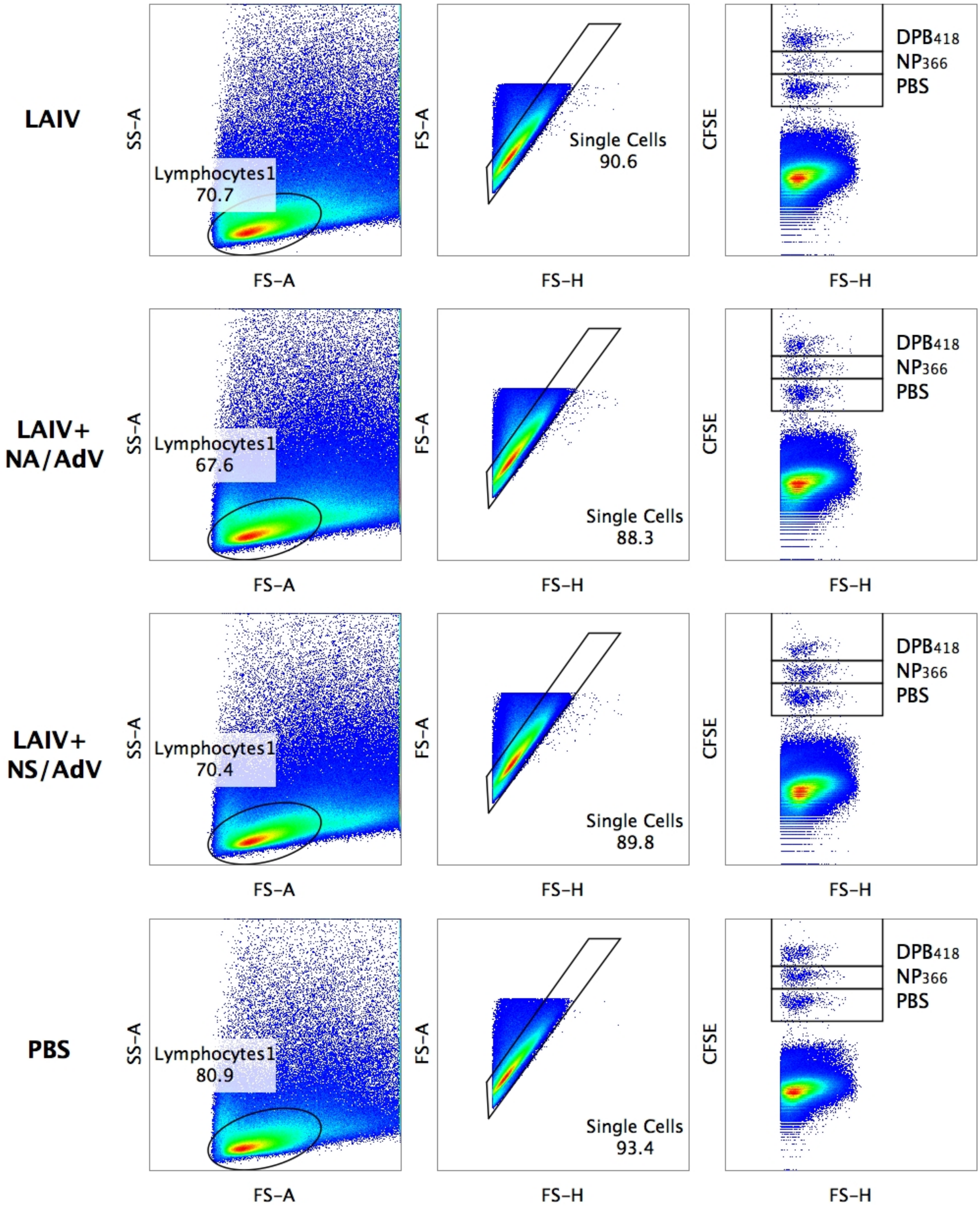


**Figure S2.** Gating strategy for CTL *in vivo* assay.

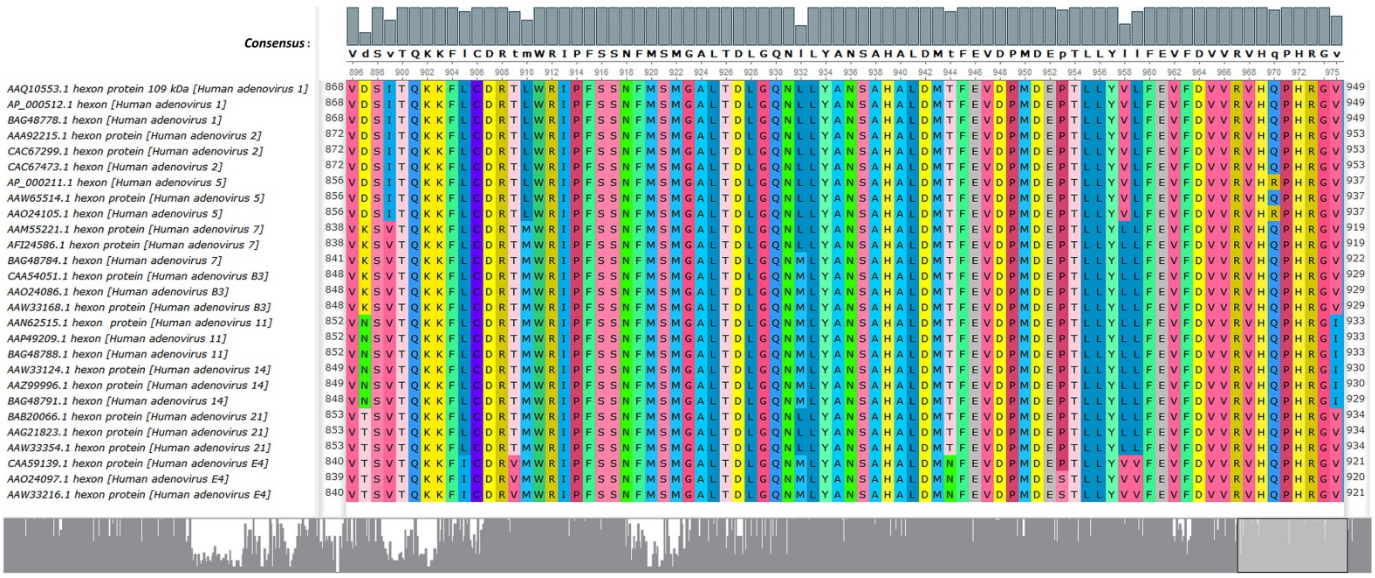

**Figure S3.** Alignment of a C-terminus fragment of AdV hexon (residues 855-935) among various AdV serotypes belonging to different AdV groups. Upper histogram shows conservancy of the amino acids within the selected fragment. Lower histogram shows the overall conservancy of the whole hexon protein, with the selected fragment depicted in the frame.

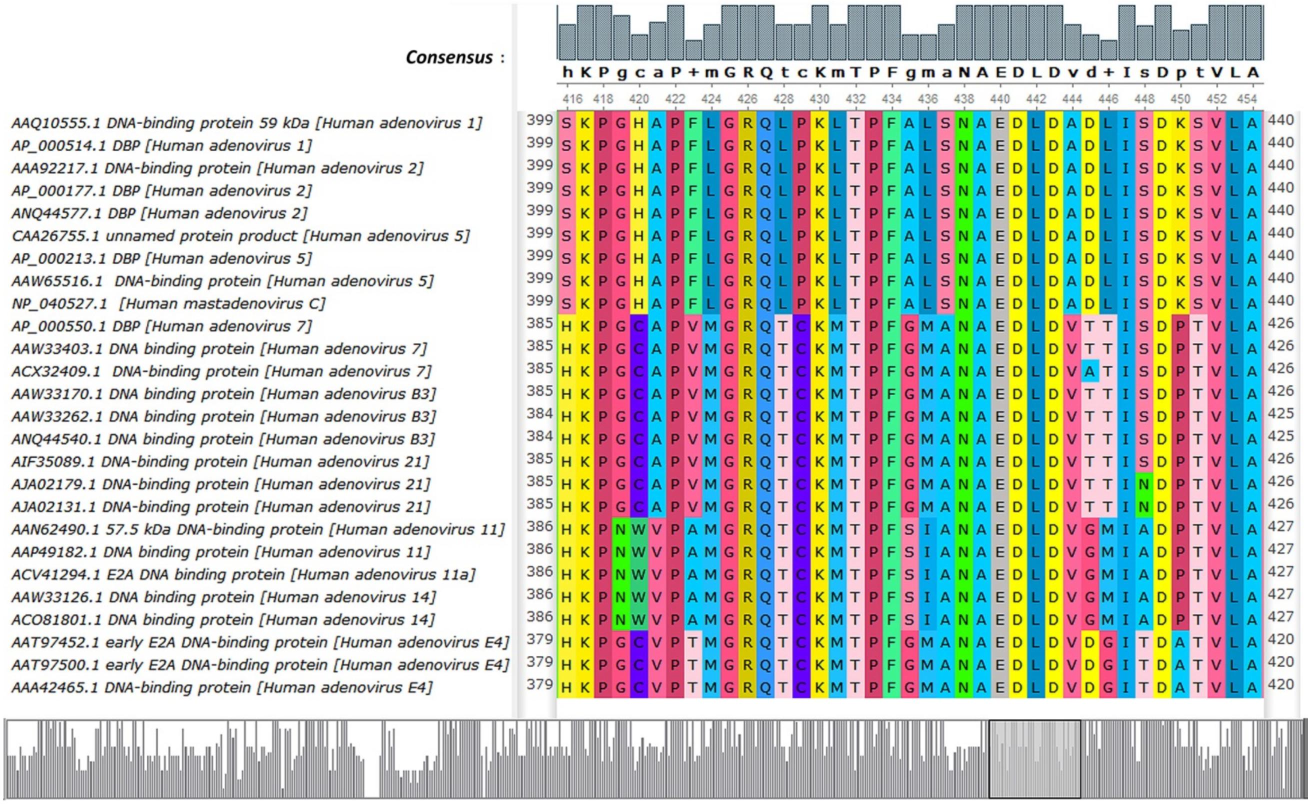

**Figure S4**. Alignment of a DBP region (residues 400-438) among various AdV serotypes belonging to different AdV groups. Upper histogram shows conservancy of the amino acids within the selected fragment. Lower histogram shows the overall conservancy of the whole DBP, with the selected fragment depicted in the frame.

## References


1. Chakupurakal G., Onion D., Bonney S., Cobbold M., Mautner V. and Moss P. HLA-peptide multimer selection of adenovirus-specific T cells for adoptive T-cell therapy. Journal of immunotherapy 36(8):423-431, 2013.
2. Comoli P., Schilham M.W., Basso S., van Vreeswijk T., Bernardo M.E., Maccario R., van Tol M.J., Locatelli F. and Veltrop-Duits L.A. T-cell lines specific for peptides of adenovirus hexon protein and devoid of alloreactivity against recipient cells can be obtained from HLA-haploidentical donors. Journal of immunotherapy 31(6):529-536, 2008.
3. Dasari V., Schuessler A., Smith C., Wong Y., Miles J.J., Smyth M.J., Ambalathingal G., Francis R., Campbell S., Chambers D. and Khanna R. Prophylactic and therapeutic adenoviral vector-based multivirus-specific T-cell immunotherapy for transplant patients. Molecular therapy. Methods & clinical development 3:16058, 2016.
4. Dorrie J., Krug C., Hofmann C., Muller I., Wellner V., Knippertz I., Schierer S., Thomas S., Zipperer E., Printz D., Fritsch G., Schuler G., Schaft N. and Geyeregger R. Human adenovirus-specific gamma/delta and CD8+ T cells generated by T-cell receptor transfection to treat adenovirus infection after allogeneic stem cell transplantation. PloS one 9(10):e109944, 2014.
5. Gerdemann U., Keirnan J.M., Katari U.L., Yanagisawa R., Christin A.S., Huye L.E., Perna S.K., Ennamuri S., Gottschalk S., Brenner M.K., Heslop H.E., Rooney C.M. and Leen A.M. Rapidly generated multivirus-specific cytotoxic T lymphocytes for the prophylaxis and treatment of viral infections. Molecular therapy : the journal of the American Society of Gene Therapy 20(8):1622-1632, 2012.
6. Gunther P.S., Peper J.K., Faist B., Kayser S., Hartl L., Feuchtinger T., Jahn G., Neuenhahn M., Busch D.H., Stevanovic S. and Dennehy K.M. Identification of a Novel Immunodominant HLA-B*07: 02-restricted Adenoviral Peptide Epitope and Its Potential in Adoptive Transfer Immunotherapy. Journal of immunotherapy 38(7):267-275, 2015.
7. Haveman L.M., Bierings M., Klein M.R., Beekman J.M., de Jager W., Kuis W., Albani S. and Prakken B.J. Selection of perforin expressing CD4+ adenovirus-specific T-cells with artificial antigen presenting cells. Clinical immunology 146(3):228-239, 2013.
8. Haveman L.M., Bierings M., Legger E., Klein M.R., de Jager W., Otten H.G., Albani S., Kuis W., Sette A. and Prakken B.J. Novel pan-DR-binding T cell epitopes of adenovirus induce pro-inflammatory cytokines and chemokines in healthy donors. International immunology 18(11):1521-1529, 2006.
9. Heemskerk B., van Vreeswijk T., Veltrop-Duits L.A., Sombroek C.C., Franken K., Verhoosel R.M., Hiemstra P.S., van Leeuwen D., Ressing M.E., Toes R.E., van Tol M.J. and Schilham M.W. Adenovirus-specific CD4+ T cell clones recognizing endogenous antigen inhibit viral replication in vitro through cognate interaction. Journal of immunology 177(12):8851-8859, 2006.
10. Imahashi N., Nishida T., Ito Y., Kawada J., Nakazawa Y., Toji S., Suzuki S., Terakura S., Kato T., Murata M. and Naoe T. Identification of a novel HLA-A*24:02-restricted adenovirus serotype 11-specific CD8+ T-cell epitope for adoptive immunotherapy. Molecular immunology 56(4):399-405, 2013.
11. Joshi A., Zhao B., Romanowski C., Rosen D. and Flomenberg P. Comparison of human memory CD8 T cell responses to adenoviral early and late proteins in peripheral blood and lymphoid tissue. PloS one 6(5):e20068, 2011.
12. Leen A.M., Christin A., Khalil M., Weiss H., Gee A.P., Brenner M.K., Heslop H.E., Rooney C.M. and Bollard C.M. Identification of hexon-specific CD4 and CD8 T-cell epitopes for vaccine and immunotherapy. Journal of virology 82(1):546-554, 2008.
13. Leen A.M., Sili U., Savoldo B., Jewell A.M., Piedra P.A., Brenner M.K. and Rooney C.M. Fiber-modified adenoviruses generate subgroup cross-reactive, adenovirus-specific cytotoxic T lymphocytes for therapeutic applications. Blood 103(3):1011-1019, 2004.

14. Leen A.M., Sili U., Vanin E.F., Jewell A.M., Xie W., Vignali D., Piedra P.A., Brenner M.K. and Rooney C.M. Conserved CTL epitopes on the adenovirus hexon protein expand subgroup cross-reactive and subgroup-specific CD8+ T cells. Blood 104(8):2432-2440, 2004.
15. Lugthart G., Albon S.J., Ricciardelli I., Kester M.G., Meij P., Lankester A.C. and Amrolia P.J. Simultaneous generation of multivirus-specific and regulatory T cells for adoptive immunotherapy. Journal of immunotherapy 35(1):42-53, 2012.
16. Olive M., Eisenlohr L., Flomenberg N., Hsu S. and Flomenberg P. The adenovirus capsid protein hexon contains a highly conserved human CD4+ T-cell epitope. Human gene therapy 13(10):1167-1178, 2002.
17. Onion D., Crompton L.J., Milligan D.W., Moss P.A., Lee S.P. and Mautner V. The CD4+ T-cell response to adenovirus is focused against conserved residues within the hexon protein. The Journal of general virology 88(Pt 9):2417-2425, 2007.
18. Tang J., Olive M., Champagne K., Flomenberg N., Eisenlohr L., Hsu S. and Flomenberg P. Adenovirus hexon T-cell epitope is recognized by most adults and is restricted by HLA DP4, the most common class II allele. Gene therapy 11(18):1408-1415, 2004.
19. Tischer S., Geyeregger R., Kwoczek J., Heim A., Figueiredo C., Blasczyk R., Maecker-Kolhoff B. and Eiz-Vesper B. Discovery of immunodominant T-cell epitopes reveals penton protein as a second immunodominant target in human adenovirus infection. Journal of translational medicine 14(1):286, 2016.
20. Veltrop-Duits L.A., Heemskerk B., Sombroek C.C., van Vreeswijk T., Gubbels S., Toes R.E., Melief C.J., Franken K.L., Havenga M., van Tol M.J. and Schilham M.W. Human CD4+ T cells stimulated by conserved adenovirus 5 hexon peptides recognize cells infected with different species of human adenovirus. European journal of immunology 36(9):2410-2423, 2006.
21. Zhu F., Xu H., Tsao A., Margolis D.A. and Keever-Taylor C.A. Generation of cytotoxic T-cell lines using overlapping pentadecapeptides derived from conserved regions of the adenovirus hexon protein. The Journal of general virology 91(Pt 6):1577-1589, 2010.
